# Serotonin transporter gene polymorphism and treatment response to serotonin reuptake inhibitor (escitalopram) in depression: An open pilot study

**DOI:** 10.4103/0019-5545.39759

**Published:** 2008

**Authors:** Mushtaq A. Margoob, Dhuha Mushtaq, Imtiyaz Murtza, Huda Mushtaq, Arif Ali

**Affiliations:** Department of Psychiatry, Govt. Medical College, Srinagar, Kashmir, India; 1 Department of Biosciences, Gene Expression Lab, Jamia Millia Islamia, New Delhi, India; 2 Department of Biochemistry and Molecular Biotechnology, Division of PHT, SKUAST, Srinagar, Kashmir, India; 3 Department of Clinical Psychology, Institute of Human Behaviour and Allied Sciences, Delhi, India

**Keywords:** Depression, serotonin transporter gene polymorphism, SSRI response

## Abstract

**Background:**

Blocking of the serotonin transporter is the main mechanism of action of SSRIs; therefore, the gene encoding this protein is a strong candidate for a possible genetic influence on the treatment response.

**Aim:**

To evaluate relationship between serotonin transporter gene promoter region polymorphism and the efficacy of SSRI (escitalopram) treatment in depression.

**Materials and Methods:**

Fifty-seven consecutive patients with unipolar depressive episode (DSM IV criteria) were genotyped for the SERT gene polymorphism and treated with escitalopram 20 mg/day. Weekly assessment (HAM-D-21) was made for treatment response up to 6 weeks.

**Results:**

Significant (*P* > 0.0001) difference between groups (ll *vs.* ss or ls) in response to treatment by escitalopram was revealed by our study. However, no difference with respect to age, gender, or onset of illness was observed between genotype subgroups.

**Conclusion:**

The study suggests that serotonin transporter gene polymorphism may have an influence on the effectiveness of SSRI treatment in depressive disorders, irrespective of clinical variables. Further controlled studies are required to validate these results

## INTRODUCTION

Though more than one and half century ago psychiatric ailments had been recognized as the disorders of the brain by researchers like Griesinger,[1] their precise etiology remains to be thoroughly clarified even today. Lack of biological markers continues to be a major impediment in the early diagnosis and effective treatment of various psychiatric disorders.

Monoamine transporters selective for dopamine, norepinephrine, and serotonin are currently the main pharmacological targets for many therapeutic agents used for the treatment of a number of psychiatric conditions. They not only limit the intensity of monoamines at the presynaptic and postsynaptic receptors but also serve as the primary mechanism for maintaining a transmitter pool ready for subsequent release.[2] Efforts have recently begun to search for polymorphic variations of transporters regulating monoamine homeostasis, to gain a better understanding into the vulnerability and pathophysiology of common psychiatric disorders like depression,[3–7] obsessive compulsive disorder,[8] posttraumatic stress disorder,[9,10] etc., alternatively providing means to prove the efficacy of clinical intervention in these disorders. Serotonin-mediated neurotransmission contributes to different functions, including motor activity, food intake, sleep and reproductive activity, as well as cognition and emotional states of depression and anxiety. The 5HT transporter (5HTT) gene product plays a key role in regulating the duration and magnitude of serotonergic responses for fine-tuning of brain serotonergic and the peripheral actions of serotonin. It provides the primary target for the action of selective antidepressant drugs in the brain. The human serotonin transporter protein is encoded by a single gene located on chromosome 17q 11.1 - 17 q12; it spans 31 kilobases (kb) and consists of 14 exons.[11] Recent studies have reported both positive and negative association between the serotonin transporter (5-HTTPR) gene regulatory region polymorphism and many psychiatric disorders, like depression,[12–14] obsessive compulsive disorder,[15,16] and posttraumatic stress disorder.[9,10]

Functionally two common alleles of serotonin transporter promoter region include short (s) allele of 448 base pairs (bp) and the long (l) allele of 528 bp. An attenuated promoter segment of (s) allele is associated with reduced transcription and functional capacity of serotonin transporter relative to (l) allele.

The aim of this study was to test for an association between SSRI treatment response in major depressive disorders and the allelic variation of the serotonin transporter gene.

## MATERIALS AND METHODS

### Subjects

The study sample comprised of first-visit 57 (21 males, 36 females) patients (mean age, 39 ± 8.3 years) of depression who sought consultation of the first author at the O.P.D. of a tertiary care hospital at the Department of Psychiatry, Government Medical College, Srinagar, or at the private clinic of the author. After the procedure was fully explained, informed consent was obtained from each subject who agreed to participate. Besides socio-demographic profile, the subjects were evaluated for DSM IV-based Axis I diagnosis for unipolar depression using structured clinical interview (SCIDI).[17] We used escitalopram 20 mg as prototype SSRI in our open-label fixed-dose study as it is the most specific serotonin transporter inhibitor compound available at present and has also a self-potentiating effect through an allosteric mechanism on the serotonin transporter.[18] To test genotype effect and treatment outcome, the 21-item Hamilton Rating[19] Scale for Depression (HAM-D-21; Hamilton 1967) was administered by trained staff. From the observed scores, predicted scores were calculated and results computed accordingly.

### Genotyping

DNA was extracted from the lymphocytes harvested from the whole blood using Bangalore Geni Kit (Indian make). Samples from 57 subjects were analyzed for 5HTT promoter region polymorphism. The insertion/deletion was checked by amplification of this region by polymerase chain reaction with oligonucleotide primers. Polymerase chain reaction was performed in Techgene thermal cycler (Techne, U.K.) with 50 ng genomic DNA and 10 pmol of each primer using PCR amplification kit (Imperial, India) in the total volume of 50 L. Cycling conditions were preceded by a denaturing step at 93°C for 5 min, followed by 35 cycles of denaturation at 94°C for 1 min, annealing at 61°C for 1 min, and synthesis at 72°C for 1 min. Allele sizes were determined by the comparison of bands with size standards after electrophoresis in 8% polyacrylamide gel followed by silver staining. Allelic distribution and the test for Hardy-Wienburg equilibrium were performed.

## RESULTS

Forty-one (72%) of the escitalopram-treated subjects completed the trial. Of the 57 subjects genotyped, 19 (33.34%) had II genotype, 25 (43.8%) had Is genotype, and 13 (22.9%) had ss genotypes. Since s allele has been demonstrated to be functionally dominant,[2] genotypes were clubbed into two groups only: those with II genotype (33.34%) and those having either ss or Is genotype (66.66%). No difference with respect to age, gender, age at onset of first episode of depression was observed between the two groups. However, the study revealed a significant (*P* < 0.001) group effect. At the third week of treatment, 12 (63.15%) of the II group had a 50% reduction in Hamilton score compared to 4 (10.52%) of the s group (*P* < 0.001). The mean percentage in HRDS scores declined - from base value, which at that time was 58.4% ± 10% for the II group as compared to 34.3% ± 5% for those with one or two s alleles. Almost the same treatment response trend continued up to the end of the sixth week.

## DISCUSSION

SSRIs as the first-line treatment today for majority of the patients of depression show a large variability in the inter-individual pharmacological response pattern. Genetic factors appear to be relevant biological determinants of treatment responses, as pedigrees with a homogenous transmission model have already been reported with similar response to the same antidepressant group in families.[4] Many recent investigations have suggested that the 5HTTLPR polymorphism has a role to play in human depression.[20] Our study also supports a relationship between serotonin transporter gene polymorphism and the efficacy of SSRIs. The short (ss) variant, as well as heterozygous Is genotype, showed a poorer response to SSRI treatment. These results are in agreement with a recent double-blind, randomized study investigating the influences of 5HTTLPR polymorphism at the *SLC6A4* locus vis-á-vis the efficacy and tolerability of mirtazapine and paroxetine, which reported that the *s* allele is associated with a poor outcome after treatment with selective serotonin reuptake inhibitors.[21]

Studies looking into the involvement of SERT in depression have not been consistent.[22] Through the modulations of serotonergic and noradrenergic systems, however, the therapeutic efficacy studies of most antidepressants continue to be linked to functional polymorphism within the promoter region of the serotonin transporter gene. It is now commonly accepted that that the 80% occupancy of the serotonin transporter with the SSRIs is therapeutically useful,[23] thus indicating that polymorphism in this gene could influence the treatment. The s allele has been postulated to manifest with higher depression scores when life events are more severe.[24] By attenuating the impact of life events, the s allele is believed to exercise both a direct and indirect effect on the severity of depression. It has also been reported recently that the serotonin transporter is allosterically modulated by some SSRIs.[25] Short variant has been reported to be associated with a poorer response to various antidepressant treatments.[26,27] However, the findings have not been supported by studies undertaken among Korean and Japanese population, which on the contrary reported that the short (s) allele was associated with more favorable treatment outcomes.[28,29] A most recently published study among Korean depressive patients has also shown that responses at the second and fourth weeks to mirtazapine treatment were significantly better for the s/s genotype of the 5-HTTLPR polymorphism than for I-allele carriers, which the authors conclude as “supporting the hypothesis that the response to noradrenergic and specific serotonergic antidepressants is significantly associated with the 5-HTTLPR polymorphism.”[30] A recent genomic analysis for the long and short allele variants of the 5HTTLPR polymorphism carried out by Ng *et al.* among two ethnic groups reported a higher rate of I/I genotype among Caucasian subjects, while Chinese subjects had higher frequencies of I/s and s/s genotypes.[31] However, comparison of the 5-HTTLPR s/s genotype subject subgroup with I/I and I/s genotype subject subgroups found no significant differences in the HDRS scores, CGI scores, response rates, adverse effects, and sertraline plasma concentrations with sertraline treatment at week 6. The negative correlation could also be the result of a methodological fallacy of comparing the I/I and I/s genotype together, with s/s genotype subgroup. Robust scientific data has suggested that attenuated promoter segment of (s) allele, whether single or in pair, is associated with reduced transcription and functional capacity of serotonin transporter relative to (I) allele. The contradictory results of SERT gene polymorphism studies among different ethnic groups and antidepressant treatment responses among allelic variants in them are beyond the scope of this article, but future genetic exploration of such variations may help to understand the underlying genetic mechanisms of such discrepancies. In one of the first allelic variation and common psychiatric disorder studies among Indian population, Mukherji *et al.* observed that serotonin transporter allele frequencies in the Indian population differed from those in the other populations.[32]

A study of association of serotonergic candidate genes and puerperal psychosis among Indian population has also been published by this group in 2007.[33]

Future functional polymorphism investigations of recently described two functionally distinct subtypes of long allele A and G may also help to clarify inconsistent findings of SERT polymorphism association studies.[31] Hu *et al.* in a recently conducted study on association between functional serotonin transporter promoter polymorphism and citalopram treatment in adult white American outpatients with major depression reported that a lesser adverse effect burden was associated with *L*_A_*L*_A_ genotype frequency or *L*_A_ allele frequency among subsample, which did not hold when the *L* allele was undifferentiated.[34] Development of diarrhea and the presence of the low-expression *S* or *L*_A_ alleles were the strongest risk factors associated with adverse effect burden. The study concludes that because the *L*A allele confers increased *SLC6A4* transcription, increased serotonin transporter levels in brain and other tissues may lead to fewer adverse effects for antidepressant medications that target the transporter.[35]

If confirmed by large, concentration-controlled studies in future, the findings may improve the efficacy of psychopharmacological intervention in depression by helping the clinician to tailor treatment according to the patient's individual genetic pattern. Such molecular genetic, multiple, variable, controlled, prospective studies, which hopefully will include additional measures, may provide better answer to the role of SERT gene in predicting antidepressant response.
